# SUMOylation and calcium signalling: potential roles in the brain and beyond

**DOI:** 10.1042/NS20160010

**Published:** 2017-07-19

**Authors:** Leticia Coelho-Silva, Gary J. Stephens, Helena Cimarosti

**Affiliations:** 1 Department of Pharmacology, Federal University of Santa Catarina, Florianopolis, Brazil; 2 School of Pharmacy, University of Reading, Reading, U.K.

**Keywords:** Ca2+ channels, neurotransmission, post-translational protein modification, SUMO, SUMOylation

## Abstract

Small ubiquitin-like modifier (SUMO) conjugation (or SUMOylation) is a post-translational protein modification implicated in alterations to protein expression, localization and function. Despite a number of nuclear roles for SUMO being well characterized, this process has only started to be explored in relation to membrane proteins, such as ion channels. Calcium ion (Ca^2+^) signalling is crucial for the normal functioning of cells and is also involved in the pathophysiological mechanisms underlying relevant neurological and cardiovascular diseases. Intracellular Ca^2+^ levels are tightly regulated; at rest, most Ca^2+^ is retained in organelles, such as the sarcoplasmic reticulum, or in the extracellular space, whereas depolarization triggers a series of events leading to Ca^2+^ entry, followed by extrusion and reuptake. The mechanisms that maintain Ca^2+^ homoeostasis are candidates for modulation at the post-translational level. Here, we review the effects of protein SUMOylation, including Ca^2+^ channels, their proteome and other proteins associated with Ca^2+^ signalling, on vital cellular functions, such as neurotransmission within the central nervous system (CNS) and in additional systems, most prominently here, in the cardiac system.

## Introduction

The small ubiquitin-like modifier (SUMO) was first described as targeting nuclear proteins that regulate transcription factors, gene expression and DNA integrity [1]. Experiments with knockout mice for the sole SUMO conjugating enzyme, ubiquitin-like conjugating enzyme 9 (Ubc9), demonstrated nuclear dysfunction and embryonic lethality, confirming that SUMOylation is physiologically indispensable [2]. Reports that are more recent have shown that SUMO can also target cytosolic and membrane proteins, including ion channels, to regulate crucial cellular functions, such as plasma membrane depolarization and neurotransmission [3,4]. So far, the majority of studies have focused on SUMOylation of potassium (K^+^) channels, which are involved in setting the duration and firing pattern of action potentials [5]. For example, SUMOylation can modulate both two-pore domain K^+^ (K2P) channels [3,6–9], responsible for the regulation of background leak currents, and voltage-dependent K^+^ (K_V_) channels [10–13] that repolarize cell membrane during action potential input. However, there is also recent evidence that voltage-gated Ca^2+^ channels (VGCCs) [14] and transient receptor potential (TRP) channels [15], both of which can mediate Ca^2+^ influx, are SUMO targets. Considering the utmost relevance of Ca^2+^ in physiological and pathophysiological processes, and the growing evidence that SUMO can modify ion channels, our review focused on the potential roles of SUMOylation of Ca^2+^ channels and proteins related with Ca^2+^ signalling with a focus on the central nervous system (CNS) and, also, the cardiac system.

## SUMOylation pathways

Post-translational modifications of proteins can affect their function, localization and degradation depending on the stimulus applied, to control cellular response [16,17]. SUMOylation is a reversible lysine-targeted post-translational modification, whereby covalently conjugated SUMO regulates proteins in numerous pathways [18,19]. Currently, there are five proposed SUMO isoforms, with SUMO-1, 2 and 3 being the best-characterized paralogs. SUMO-1 shares approximately 50% of its amino acid sequence with both SUMO-2 and SUMO-3, which are typically known as SUMO-2/3 since they differ by only three N-terminal amino acids and antibodies are usually unable to distinguish between them [20,21]. Despite the similarities, there are functional differences between SUMO-1 and SUMO-2/3. For instance, under basal conditions, unconjugated SUMO-1 is scarce, but free SUMO-2/3 is widely expressed in mammalian cells [22]. Although the exact role for SUMO-4 remains uncertain, it has been associated with the pathophysiological mechanisms underlying diabetes [23,24]. Finally, the existence of a fifth SUMO isoform, SUMO-5, that regulates promyelocytic leukaemia nuclear bodies, has recently been suggested [25]. The same enzymes conjugate all SUMO isoforms [19].

The first step in the SUMOylation process requires the maturation of SUMO by SUMO-specific isopeptidases/proteases; next, SUMO is activated in an ATP-dependent step by E1 complex, which in humans consists of an heterodimer formed by SUMO-activating enzyme subunits 1 and 2 (SAE1 and SAE2 respectively). Subsequently, SUMO is transferred from the E1 activating enzyme to the E2 conjugating enzyme, also known as Ubc9, which is able to conjugate SUMO to target proteins both in E3 ligase-dependent and -independent manners. Most target proteins carry the same consensus motif that is directly recognized by Ubc9: the –K–x–D/E sequence, with representing a large hydrophobic residue (commonly isoleucine, leucine or valine), K is the modified lysine, x is any residue and D/E are acidic residues [22,26]. Nevertheless, non-covalent interactions between SUMO and target proteins can occur through SUMO interacting motifs (SIMs) [17,27]. These SIMs consist of a short stretch of branched hydrophobic residues, typically comprising isoleucine (I) or valine (V) residues organized as (V/I)–x–(V/I)–(V/I) or (V/I)–(V/I)–x–(V/I), flanked NH_2_– or COOH– terminally by serine residues and/or acidic residues [28]. Alternatively, SUMO E3 ligases can directly bind to target proteins [17]. The SUMOylation process is highly reversible by the same enzymes responsible for SUMO maturation and also SUMO deconjugation from substrate proteins [29].

Recently, three distinct families of SUMO-specific isopeptidases and proteases have been identified in mammals: the ubiquitin-like protease/sentrin-specific protease (Ulp/SENP), the deSUMOylating isopeptidase (Desi) and ubiquitin-specific peptidase-like protein 1 (USPL1) [30,31]. The SENPs are the best characterized and, so far, six SENP isoforms have been identified in humans: SENP1, 2, 3, 5, 6 and 7 [17]. SENP1 is highly expressed in the nucleus, in the nuclear pore and as discrete nuclear ‘dots’ [32], but can also be found in all neuronal processes and at synapses at lower levels [33–35]. During the maturation phase, SENP1 cleaves pro-SUMO preferentially to generate SUMO-1 and SUMO-2/3 [36,37], while it deconjugates both SUMO isoforms [37,38]. SENP2 is similar to SENP1 with respect to its localization and characteristics regarding the maturation step, but differs from SENP1 regarding its highly selectivity for SUMO-2/3 deconjugation [37–40]. SENP3 is found in the nucleus, but also in the mitochondria and participates in neuronal signalling [41]. The role of SENP3 in cleaving pro-SUMO has not been elucidated as yet, but it is suggested that SENP3 is somehow selective for removing SUMO-2/3 from target proteins [37,38]. As for SENP3, SENP5 has a nuclear localization [37,42] and is important for SUMO-2/3 maturation and deconjugation [37,38,43]. Finally, SENP6 and SENP7 are located throughout the nucleoplasm [17,44] and, although neither participates in the maturation step, they are both important for removal of SUMO-2/3 [17,44,45]. Regarding the Desi family, two isoforms have been identified so far: Desi-1 and Desi-2. Whereas Desi-1 is found both in the cytoplasm and the nucleus, where it promotes deconjugation of all SUMO isoforms, Desi-2 is exclusively cytoplasmatic and its properties remain undefined [30,31]. Lastly, USPL1 preferably promotes SUMO-2/3 deconjugation and is located in Cajal bodies [30,31].

## Roles of SUMOylation in neurological diseases

Disruption of basal SUMOylation has been implicated in multiple neurological disorders, including neurodegenerative diseases, such as Alzheimer and Parkinson’s diseases (AD and PD respectively), spinocerebellar ataxias (SCAs), cerebral ischaemia and epilepsy [46]. More specifically, amyloid precursor protein (APP) and tau, which are key proteins in AD, have been identified as SUMO targets in HeLa and HEK293 cells [47–49]. APP undergoes proteolytic cleavage by α- or β-secretases, and both are followed by further γ-secretase processing [50]. While α-secretases cleave APP to peptides that are proposed to participate in neuroprotection and neuroplasticity, characterizing the non-amyloidogenic pathway [51], cleavage by β-secretases leads to the amyloidogenic pathway, generating toxic amyloid β (Aβ) that accumulates and forms amyloid plaques [52]. A reduction in Aβ aggregates was found in HeLa cells when APP was SUMOylated by either SUMO-1 or SUMO-2 at lysines 587 and 595, which are located adjacently to the β-secretase site [48]. Moreover, poly-SUMOylation of APP by SUMO-3 has been reported to regulate APP cleavage and decrease Aβ production in HEK293 cells [53]. Conversely, SUMO-3, as well as SUMO-1, was found to increase γ-secretase levels [54], thus increasing Aβ production in a transgenic mice model for AD [55]. It is important to note that SUMO-3 effects on Aβ deposition might not be dependent on the ability of SUMO-3 to conjugate to target proteins [54]. Another AD hallmark is the hyperphosphorylation of tau [56] that decreases its affinity for microtubules, resulting in tau accumulation and formation of neurofibrillary tangles [57]. Tau can undergo SUMOylation at lysine 340 in HEK293 cells, which triggered its phosphorylation and inhibited its degradation by the ubiquitin–proteasome pathway, thus increasing tau aggregation [47].

As for mouse models of AD [55], increased levels of SUMO-1 were found in the plasma of patients with dementia [58]. Conversely, SUMO-1 conjugates were not altered in the post-mortem hippocampus of AD patients, whereas SUMO-2/3 high molecular weight conjugates were decreased [59]. These observations are in agreement with previous reports that found increased SUMO-1 and decreased SUMO-2 conjugation levels in the cortex and hippocampus respectively, of Tg2576 mice [60,61]. However, a recent study demonstrated absence of gross changes in global SUMOylation levels in the post-mortem cortex of AD patients [62].

α-Synuclein, parkin and DJ-1 are examples of SUMO targets relevant to PD [17,63,64]. Cytosolic inclusions known as Lewy bodies, comprised mostly by aggregated α-synuclein, contribute to the synaptic dysfunction and consequent dopaminergic neuronal death predominantly in the substantia nigra, a well-described characteristic of PD [65–68]. Promisingly, SUMO-1 conjugation to α-synuclein reduced its aggregation and toxicity in a transgenic mice model for PD [69]. Interestingly, in an early communication, lysosomal SUMO-1 labelling was identified in human olfactory mucosa-neurospheres obtained from biopsies of patients with idiopathic PD [70]. A similar finding was observed in post-mortem tissue from patients with multiple system atrophy and progressive supranuclear palsy, diseases in which α-synuclein and tau seem to be involved [70,71]. In both familial and sporadic PD, parkin, which is an ubiquitin ligase, can be found together with α-synuclein in Lewy bodies, where SUMO-1 was shown to non-covalently and selectively interact with parkin, increasing its auto-ubiquitination and transportion to the nucleus [72]. Moreover, SUMOylation of DJ-1, a transcriptional regulator mutated in 1–2% of early-onset PD cases, maintained its cytoprotective function in response to oxidative stress [73,74], whereas incomplete SUMOylation of DJ-1 led to its proteasomal degradation [75]. In a similar way to SUMOylated α-synuclein, increased SUMO conjugation to ataxin-7 decreased its aggregation and cytotoxicity in SCAs [76].

Despite several reports from our group and others showing that SUMOylation can protect cells from metabolic stress caused by low levels of oxygen and glucose in different models of cerebral ischaemia and hypoxic conditions [77–81], disease-modified SUMO targets remain largely unknown. However, one such target is the mitochondrial GTPase dynamin-related protein 1 (Drp1), which regulates mitochondrial fission [41,82]. Under stress conditions, Drp1-mediated mitochondrial fission can release cytochrome *c* and induce caspase cleavage followed by cell apoptosis [83]. In an *in vitro* model of ischaemia, oxygen and glucose deprivation led to SENP3 degradation and consequent increase in SUMO-2/3 conjugation to Drp1, thus preventing mitochondrial fission and cytochrome *c* release, as well as promoting cell survival [41]. Another ischaemia-modified SUMO target is the isoform 3 of the sodium (Na^+^)/Ca^2+^ exchanger (NCX), which controls ionic homoeostasis during cerebral ischaemia [84]. NCX3 f-loop lysine 590 is required for SUMOylation, and the absence of this residue increased NCX3 degradation, exacerbating ischaemic damage induced by permanent and transient middle cerebral artery occlusion (MCAO) [85]. Following preconditioning and transient MCAO, SUMO-1 basal expression led to increased NCX3 levels, whereas SUMO silencing decreased NCX3 levels, suggesting that NCX3 SUMOylation participates in the protective role that SUMO-1 plays during ischaemic preconditioning [85].

Evidence shows that SUMOylation may be involved in mechanisms implicated in the development and maintenance of epilepsy, since it was demonstrated that neuronal K^+^ channels could be SUMOylated, thus modulating neuronal excitability [3,6–10]. Moreover, SUMOylation of excitatory receptor subunits can modulate receptor trafficking and interfere with synaptic transmission [86–90]. For example, SUMOylation of the GluK2 subunit of kainate receptors led to receptor internalization, which could be neuroprotective against excitotoxicity [33]. More recently, the major cause of premature death in epilepsy, known as sudden unexplained death in epilepsy, has been linked with the hyper-SUMOylation of the K_V_7 K^+^ channel, which functionally reduces the depolarizing M-current conducted by this channel [13].

## Ca^2+^ channels

Unique amongst other ions, Ca^2+^ can modulate both membrane potential and function as an important signalling entity. Several cellular processes, ranging from neurotransmitter/hormone release [91] and muscle contraction [92] to gene transcription [93,94], require an increase in the intracellular Ca^2+^ levels, which under basal conditions are maintained approximately 100 nM [95]. This temporary increase occurs by either release from intracellular Ca^2+^ stores or influx into the cell by agonist-operated channels, G-protein coupled receptors, store-operated channels and, predominantly, through VGCCs located at the plasma membrane [96].

VGCCs were initially classified based on their voltage-dependent activation (high or low voltage-activated channels) [97,98] and subsequently subdivided by pharmacological and biophysical function (high voltage-activated and low voltage-activated) [99] and then by Ca_V_α1 subunits [100]. Ca_V_α1 structure allows selectivity for Ca^2+^ over monovalent ions and contains a sensor motif that detects membrane depolarization leading to channel opening [96]. Based on their Ca_V_α1 subunits, three families of VGCCs have been defined: Ca_V_1 – present mainly in skeletal muscle, heart, neurons and endocrine cells, Ca_V_2 – found mainly at presynaptic terminals in the CNS, but also in peripheral synapses, and Ca_V_3 – localized mainly in the sinoatrial node, adrenal glomerulosa cells, neurons and sperm acrosome [100,101]. Ca_V_1 subunits form L-type Ca^2+^ current; Ca_V_2.1 forms P/Q-type, Ca_V_2.2 N-type and Ca_V_2.3 form R-type current, whereas Ca_V_3 subunits form T-type current. In addition to the three Ca_V_α1 family subunits (Ca_V_1, Ca_V_2 and Ca_V_3), there are auxiliary β, α2, δ and also γ subunits that comprise the channel complex and have various functions including transporting channels from the endoplasmic reticulum to the plasma membrane, maintaining channel stability and contributing to physiological and pharmacological properties [100].

## Roles of Ca^2+^ channels in neurological disorders

Pathological changes in Ca^2+^ homoeostasis and deregulation of Ca^2+^ channels are implicated in a range of neurological disorders, including epilepsy, cerebral ischaemia, pain, neurodegenerative, and psychiatric diseases [102–104]. Ca^2+^ levels control neuronal hyperexcitability and mutations in VGCCs have been identified in familial CNS diseases (so-called ‘channelopathies’). For example, Ca_V_2.1 and Ca_V_3.2 channelopathies have been widely associated with forms of absence epilepsy and episodic ataxia [105]. Furthermore, acquired epilepsy and cerebral ischaemia can occur due to insults resulting from increased Ca^2+^ influx [105,106]. Moreover, exocytosis of synaptic vesicles mediated by VGCCs, whereby membrane depolarization triggered by action potentials causes transmitter release, may be targeted in pain pathways, in particular at central terminals of sensory nociceptive afferents. For example, both Ca_V_2.2 and Ca_V_3.2 channels are crucial for control of neurotransmitter release at the dorsal horn [107,108]. Ca_V_2.2 is targeted therapeutically by ziconotide [109,110], a drug used to treat cancer-derived pain, and other drugs targeting Ca_V_2.2 are in development [96]. Ca_V_3.2 also acts to regulate afferent fibre excitability [111] and there is good evidence that these channels are up-regulated under chronic pain conditions [112–115].

Neurodegenerative diseases and psychiatric disorders have been related to Ca^2+^ handling often with respect to mitochondrial function, since rises in Ca^2+^ levels lead to mitochondrial stress and generation of reactive oxygen species [96]. In AD, deregulation of Ca^2+^ homoeostasis contributes to Aβ production and accumulated Aβ interferes with Ca^2+^ influx. Under physiological conditions, Ca^2+^ entry is reported to contribute to APP cleavage by α-secretase, while improper intracellular Ca^2+^ mobilization can affect APP processing and lead to increased Aβ levels, neuroinflammation and metabolic stress [115,116]. Aβ is proposed to modulate Ca^2+^ influx in various ways including: by direct effects of oligomeric Aβ on the Ca_V_α1 subunit [117,118], inducing membrane-associated oxidative stress or contributing to excitotoxicity [116,119]. Moreover, mutations in Ca_V_1.2 and Ca_V_β2 have been linked to both bipolar disorder and schizophrenia, while mutations in Ca_V_1.3 have also been linked to bipolar disorder [96]. In addition, Ca_V_1.3 contributed to neuronal loss in PD as a consequence of inherent voltage-dependent activation of the subunit, rather than their selectivity for Ca^2+^ [120]. Moreover, α-synuclein aggregation can modulate the influx of Ca^2+^, and, in turn, increases in Ca^2+^ concentration can promote α-synuclein aggregation [121,122].

## SUMOylation and Ca^2+^ signalling in neurotransmission

**Figure 1:**
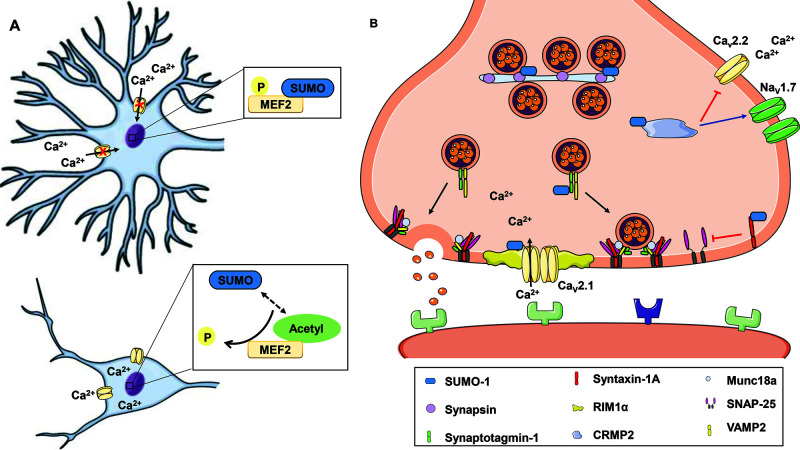
Potential roles played by SUMO on Ca^2+^ signalling in neurotransmission (**A**) Decreased calcium signalling leads to phosphorylation and SUMOylation of MEF2A, thus promoting synapse formation. As a result of VGCC activation, MEF2 is dephosphorylated and switches SUMOylation to acetylation inhibiting synaptic processes. (**B**) SUMOylated RIM1α facilitates the clustering of Ca_V_2.1 Ca^2+^ channels and enhances Ca^2+^ influx necessary for vesicular release. When SUMO is conjugated to CRMP2, it inhibits Ca^2+^ entry through Ca_V_2.2 channels, and increases surface expression of Na_V_1.7 channels. SUMOylation of syntaxin-1A, synaptotagmin-1 and synapsin la can regulate neurotransmission by participating in docking/priming of synaptic vesicles; CRMP2, collapsin response mediator protein 2; MEF2, myocyte enhancer factor 2.

SUMOylation of proteins involved in Ca^2+^ signalling affects the maintenance of neurotransmission from synapse formation (Figure 1A) to neurotransmitter release (Figure 1B) and synaptic plasticity. Mutations in the *CACNA1A* gene, which encodes the Ca_V_2.1 subunit, are found in SCA type 6 (SCA6) and lead to impaired VGCC function [123]. In an early communication, SUMO-1 overexpression was reported to decrease wild-type Ca_V_2.1 current density in HEK293 cells, whereas it had no effects on SCA6 Ca_V_2.1 mutants [124]. Interestingly, either SUMO-1 overexpression or SENP1 silencing enhanced cAMP-dependent exocytosis and glucagon secretion from both mouse and human pancreatic α-cells via effects on Ca_V_1 channels [14].

Increased SUMO-1 conjugation to presynaptic target proteins was shown to regulate Ca^2+^ influx and neurotransmitter release in synaptosomes [125]. Depending on the applied stimulus, SUMOylation of presynaptic proteins could either increase or decrease neurotransmitter release. For example, loading synaptosomes with SUMO-1 and SENP1 peptides decreased and increased Ca^2+^ influx and KCl-evoked glutamate release respectively. Conversely, kainate-induced Ca^2+^ influx and neurotransmitter release were increased in synaptosomes loaded with SUMO-1 and decreased in synaptosomes loaded with SENP1 [125]. These results suggest that SUMO may be conjugated to distinct presynaptic proteins and act in an activity-dependent and stimulus-specific manner to modulate presynaptic release.

Crucial proteins in neurotransmitter release, CRMP2 and Rab3a-interacting molecule (RIM) have been identified as members of the Ca_V_2 proteome [126]. SUMOylation of VGCC interacting proteins has been reported to play an important role in neurotransmission within pain pathways. CRMP2 interacts with Ca_V_2.2 subunits in sensory neurons or nociceptors to modulate neurotransmitter release [127]. SUMO-1–3 modified CRMP2 at lysine 374 in cultured cathecholamine A differentiated cells [128]. Overexpression of SUMO, Ubc9 and CRMP2 in adult dorsal root ganglion neurons decreased, whereas overexpression of non-SUMOylatable CRMP2 increased, KCl depolarization-induced Ca^2+^ entry. In addition, CRMP2 SUMOylation increased surface expression of Na_V_1.7 channels [129]. Mutations in Na_V_1.7 channels, which are highly expressed in peripheral sensory neurons, where they are responsible for regulating neuronal excitability, are directly related with pain disorders [130].

RIM1α interacts either directly or indirectly with most presynaptic active zone proteins and participates in the docking and priming of synaptic vesicles [131] by modulating Ca^2+^ influx through regulation of VGCCs clustering [132,133]. SUMO-1 conjugation to RIM1α at lysine 502 was shown to be crucial for normal presynaptic exocytosis in neurons [133]. Knockdown of endogenous RIM1α, and its replacement with a non-SUMOylatable mutant, led to impairment of Ca^2+^-induced depolarization and consequent removal of the fast component of vesicle exocytosis. SUMOylated RIM1α facilitated the clustering of Ca_V_2.1 channels and enhanced Ca^2+^ influx necessary for vesicular release, whereas de-SUMOylated RIM1α participated in the docking/priming of synaptic vesicles and structural maintenance of the active zone [133].

Presynaptic soluble *N*-ethylmaleimide sensitive factor attachment protein receptors (SNARE) proteins, such as syntaxin 1, are fundamental for neurotransmitter release [134] and might also participate in vesicle endocytosis [135,136]. Syntaxin 1A can be modified by SUMO-1 at any of three lysine residues (K252, K253 or K256) near the C-terminal transmembrane domain [137]. Preventing syntaxin 1A SUMOylation reduced its interaction with other SNARE proteins and disrupted the balance of synaptic vesicle endo/exocytosis, resulting in increased endocytosis. Another key protein that is SUMOylated is synapsin Ia: preventing SUMO-1 conjugation to synapsin Ia at lysine 687 caused impaired exocytosis due to a reduction in the number of releasable synaptic vesicles [138]. Proteomic analysis from a neuron-specific SUMO-1 overexpressing transgenic mouse model led to the identification of a number of previously unrecognized SUMO-1 targets *in vivo*, including the Ca^2+^ sensor synaptotagmin-1 [139]. Increased SUMO-1 conjugation to synaptotagmin-1 resulted in impaired performed paired pulse facilitation (PPF), which involves the facilitation of neurotransmitter release caused by residual Ca^2+^ from a previous stimulus.

Homologs of the SUMOylation machinery were identified in *Drosophila*, and an interaction with Ca^2+^/calmodulin-dependent protein kinase II (CaMKII) that modulates synaptic plasticity by regulating glutamatergic synapses [140] was demonstrated by yeast two-hybrid screening [141]. *Drosophila* SUMO-1 (DmSUMO-1) modification has potential to change the subcellular localization of CaMKII, but the functional consequences for this interaction remain to be confirmed.

Dendritic claws in cerebellar granular neurons, in which mossy fibre terminals and Golgi neurons form synapses [142], are regulated by the myocyte enhancer factor 2A (MEF2A). MEF2A transcription factor activity is regulated by several post-translational protein modifications, including phosphorylation [143–145], ubiquitination [146] and SUMOylation [147]. Lack of Ca^2+^ signalling led to phosphorylation of MEF2A at serine 408, which in turn led to SUMO-1 conjugation at lysine 403 and inactivation of MEF2A, promoting dendritic claw differentiation, synapse formation and maturation. Activity-dependent Ca^2+^ signalling via Ca_V_1 VGCCs induced calcineurin-mediated dephosphorylation of MEF2A at serine 408, promoting a switch from SUMOylation to acetylation at lysine 403, which in turn activated MEF2A and inhibited dendritic claw differentiation and synapse formation [147].

As previously described, deregulation of Ca^2+^ homoeostasis contributes to aggregation of proteins such as Aβ and α-synuclein, known as aggregation-prone proteins, which can interfere with neurotransmission. Also, production and accumulation of these proteins interfere with Ca^2+^ influx [148]. Two lysines of APP can be modified by SUMO *in vivo* leading to decreased levels of Aβ aggregates [48]. SUMOylation of α-synuclein seems to inhibit α-synuclein aggregation and toxicity both *in vitro* and *in vivo* [149]. This inhibition depends on the SUMO isoform (SUMO-1 conjugation is better than SUMO-3) and on the SUMOylated lysine (K102 is better than K96) [150]. Interestingly, raised concentrations of monomeric α-synuclein in the extracellular medium promoted dopamine release in the striatum via Ca_V_2.2 channels *in vivo* and *in vitro*, modifying plasma membrane structure and altering raft partitioning of this channel, suggesting the early reorganization of synaptic terminals as the mechanism to sensitizing dopaminergic neurons [151]. Paradoxically, SUMOylation of α-synuclein promoted its aggregation in COS-7 cells and had an intriguing protective effect [152].

## Roles of SUMOylation outside the brain and effects of SUMO on other channels

Other than the brain, SUMOylation is well characterized in the heart. Both Ubc9 inhibition and SUMO-2 knockout caused early embryonic lethality in mice [2,153], whereas SUMO-1 knockout led to specific cardiac septal defects [154]. Activating the SUMOylation pathway can also evoke cardiac abnormalities, such as cardiac specific SUMO-2 overexpression that induced premature death and severe cardiomyopathy [155]. Conversely, SUMO-1 overexpression improved heart failure [154–156], suggesting that tightly regulated SUMOylation levels are essential for normal cardiac development [154,157].

SUMOylation also influences cardiac metabolism, controlling crucial proteins for the maintenance of cardiac energy homoeostasis and mitochondrial biogenesis, such as peroxisome proliferator-activated receptor (PPAR) and its associated co-regulators [158]. Similarly, under metabolic stress conditions, increased cellular SUMOylation (mainly by SUMO-2/3) can protect the brain during ischaemia or hibernation torpor [158–160]. Both in animal models and human patients, a fine balance between SUMO conjugation/deconjugation is critical for cardiac stress adaptation [155,156,161,162].

SUMOylation is not only essential for cardiac development, predominantly by regulating transcription factors, but also implicated in the onset of cardiac diseases [163–165]. Several K^+^ channels found in the heart can be modulated by SUMO, such as K_V_2.1 [11,12], a channel that helps set the cell resting potential [166]; K_V_1.5 [10], which controls excitability of atrial cells [167]; and K2P1 [3,6–9], which helps set resting membrane potential. SUMOylation also regulates the cardiac non-selective cationic channel TRPM4, which is localized predominantly in human atrial myocardium, and can act as a Ca^2+^ regulator [15,168]. Progressive familial heart block type I, an autosomal dominant disease, has been linked to a mutation in the TRPM4 amino-terminal region that leads to increased TRPM4 SUMOylation and prevention of its ubiquitination and consequent proteasomal degradation [15]. Other proteins crucial for the maintenance of cardiomyocyte physiology, such as lamin A that plays a structural and functional role in the nucleus, are also reported to be SUMOylated [169,170]. Familial cardiomyopathy has been linked with mutations in the human laminin A gene, which were in turn associated with decreases in laminin A SUMOylation and accelerated cell death [169].

Disrupting Ca^2+^ dynamics by interfering with other proteins or transcriptional factors that maintain Ca^2+^ homoeostasis, such as some of TRP protein Ca^2+^ entry channels or N-terminal serine residues of the nuclear factor of activated T cells (NFAT), can contribute to the onset of cardiac dysfunctions [171]. Increased intracellular Ca^2+^ levels activate calcineurin, a Ca^2+^-calmodulin dependent serine–threonine protein phosphatase that dephosphorylates NFATs, leading to nuclear translocation of NFATs and activation of pro-hypertrophic genes [172]. SUMO-2 can activate calcineurin-NFAT signalling in cardiomyocytes leading to a hyperthophic phenotype, both *in vitro* and *in vivo* [173]. Unexpectedly, a conjugation-deficient SUMO-2 mutant (SUMO-2ΔGG) was equally capable to activate the pathway and promote hypertrophic effects, suggesting a SUMOylation-independent mechanism.

Proteins such as sarcoendoplasmic reticulum calcium ATPase (SERCA) in the sarcoplasmic reticulum and NCX in the cardiomyocyte membrane help to restore Ca^2+^ concentrations at baseline following contraction [174]. The reduced expression or activity of SERCA2a is a hallmark of heart failure [175]. A proteomic screen has identified SERCA2a as a target for SUMO-1 (but not SUMO-2/3) at lysines 480 and 585 [156]. SUMO-1 and SERCA2a protein levels were decreased in animal models of heart failure, as well as in human cardiomyocytes isolated from failing ventricles. SUMO-1 overexpression restored SERCA2a levels, whereas either SUMO-1 or SERCA2a overexpression improved Ca^2+^ handling, improving cardiac function. However, increased global SUMOylation in SERCA2a knockdown cardiomyocytes did not prevent contractile dysfunction, further confirming that SUMOylated SERCA2a is essential for cardiac function [156]. The small molecule N106 (*N*-(4-methoxybenzo[d]thiazol-2-yl)-5-(4-methoxyphenyl)-1,3,4-oxadiazol-2-amine) was identified using an α-screen assay that detects SUMO-1 conjugation to nuclear RanGAP1 (the first and one of the most stable SUMO targets identified so far [176]). N106 promoted SERCA2a SUMOylation, resulting in enhanced contractility both in cultured cardiomyocytes and *in vivo*, significantly improving ventricular function in mice with heart failure [177]. N106 was proposed to directly activate the SUMO-activating enzyme [177].

## Concluding remarks

Both alterations in Ca^2+^ homoeostasis and protein SUMOylation may lead to severe neurological, and also, cardiac pathologies. For example, SUMOylation of proteins involved in Ca^2+^ signalling can modulate synapse formation and alter neurotransmitter release. Furthermore, SUMOylation of proteins can modulate Ca^2+^ reuptake in cardiomyocytes and thus affect contractility. As described above and summarized in Table 1, it is clear that a wide range of proteins involved in these key physiological processes are subject to, potentially temporal, post-translational modification by different SUMO isoforms. Thus, at the presynapse, proteins involved in Ca^2+^ homoeostasis, including VGCCs and their proteome, are emerging as SUMO targets; equally, synaptic proteins involved in exocytosis and endocytosis are known to be SUMOylated. Postsynaptic receptor SUMOylation can also impact synaptic function. There is clear potential to exploit this knowledge to improve synaptic function in neurodegenerative and hyperexcitability disorders and to improve cardiac function. Thus, understanding how SUMOylation affects Ca^2+^ signalling in physiological and pathophysiological conditions is key to novel therapeutic strategies to prevent and/or cure important human diseases.

**Table 1:** Potential functional consequences of SUMOylation in Ca^2+^ signalling

Target (direct or indirect)	SUMO isoform	Modified lysine	Mechanism or Ca^2+^ channel type	Proposed SUMOylation effect	Reference
Ca_V_2.1 subunit (indirect)	SUMO-1	Unknown	Inhibition of P/Q-type Ca^2+^ channels	Role in SCA6 pathogenesis	[124]
CAMKII (indirect)	SUMO-1	Unknown	–	Differentiation of *Drosophila*’s nervous system	[141]
CRMP2 (direct)	SUMO-1 SUMO-2/3	K374	Inhibition of N-type Ca^2+^ channels	Reduces Ca^2+^ influx in sensory neurons	[128]
MEF2 (direct)	SUMO-1	K403	–	Promotes dendritic claw differentiation	[145,147]
NCX3 (direct)	SUMO-1	K590	–	Inhibits NCX3 degradation	[85]
NFAT (indirect)	SUMO-2	Unknown	–	Activates pro-hypertrophic genes	[173]
RIM1α (direct)	SUMO-1	K502	Increase in P/Q-type Ca^2+^ channel activity	Promotes synaptic vesicles release	[133]
SERCA2a (direct)	SUMO-1	K480 and K585	–	Increases Ca^2+^reuptake to sarcoendoplasmic reticulum	[156,177]
Synapsin Ia (direct)	SUMO-1	K687	–	Sets up releasable synaptic vesicles	[138]
Synaptotagmin-1 (indirect)	SUMO-1	Unknown	–	Impairs neurotransmitter release	[139]
Syntaxin 1A (direct)	SUMO-1	K252, K253 or K256	–	Increases vesicular endocytosis	[137]

Abbreviations: CAMKII, Ca^2+^/calmodulin-dependent protein kinase II; CRMP2, collapsin response mediator protein 2; MEF2, myocyte enhancer factor 2; NCX3, isoform 3 of the Na^+^/Ca^2+^ exchanger; NFAT, N-terminal serine residues of the nuclear factor of activated T-cells; RIM1α, Rab3a-interacting molecule 1α; SERCA2a, isoform 2a of sarcoendoplasmic reticulum Ca^2+^ ATPase.

## Abbreviations

Aβ: amyloid β
AD: Alzheimer's disease
APP: amyloid precursor protein
CNS: central nervous system
CRMP2: collapsin response mediator protein 2
Desi: deSUMOylating isopeptidase
DJ-1: PD (autosomal recessive, early onset) 7
DmSUMO-1: *Drosophila* SUMO-1
Drp1: dynamin-related protein 1
MEF2A: myocyte enhancer factor 2A
NCX: sodium/calcium exchanger
NFAT: N-terminal serine residues of the nuclear factor of activated T cells
PD: Parkinson’s disease
PPAR: peroxisome proliferator-activated receptor
PPF: paired pulse facilitation
RIM: Rab3a-interacting molecule
SCA: spinocerebellar ataxia
SERCA: sarcoendoplasmic reticulum calcium ATPase
SIM: SUMO interacting motif
SNARE: soluble *N*-ethylmaleimide sensitive factor attachment protein receptors
SUMO: small ubiquitin-like modifier
TRP: transient receptor potential
TRPM4: transient receptor potential cation channel subfamily M member 4
Ubc9: ubiquitin-like conjugating enzyme 9
USPL1: ubiquitin-specific peptidase-like protein 1
VGCC: voltage-gated calcium channel
